# Halting cyst progression in ADPKD using long-term ketogenic metabolic therapy and supplementation with exogenous ketones and alkaline citrate—a case series

**DOI:** 10.3389/fnut.2026.1843178

**Published:** 2026-06-29

**Authors:** Melina Messing, Emily Muensterman, Serena Marcon, Thomas Weimbs

**Affiliations:** 1 Department of Molecular, Cellular, and Developmental Biology, University of California Santa Barbara, Santa Barbara, CA, United States; 2 Santa Barbara Nutrients, Inc., Santa Barbara, CA, United States

**Keywords:** chronic kidney disease, exogenouse ketones, ketogenic metabolic therapy, nutrition, polycystic kidney disease

## Abstract

ADPKD is a progressive cystic disorder characterized by increasing total kidney volume (TKV), declining kidney function, and limited disease-modifying options. Emerging preclinical and clinical evidence suggests metabolic interventions may influence cyst growth, but longitudinal imaging data in humans remain limited. We conducted a retrospective case series of four self-referred individuals (ages 33–71) with genetically confirmed truncating PKD1 variants who independently implemented metabolic interventions of varying duration and intensity, including carbohydrate restriction, intermittent fasting, and supplementation with a ketone- and citrate-based medical food (KetoCitra^®^). Intervention periods ranged from 6 months to approximately 4 years and followed prior phases of documented kidney volume enlargement. Serial abdominal imaging obtained during routine clinical care was re-analyzed using standardized volumetric software to assess TKV, liver volume, and Mayo imaging classification trajectories. Across all four cases, stabilization or reduction in TKV relative to prior growth trends was observed during intervention periods, accompanied by preserved kidney function. One participant transitioned from Mayo Class 1C to 1B, while the remaining three demonstrated downward shifts in Mayo imaging trajectory within their existing classifications. Reductions or attenuated increases in liver volume were also observed in cases with polycystic liver disease. Interventions were reported to be feasible and well tolerated, with no serious adverse events. These observations align with emerging preclinical and clinical evidence suggesting metabolic interventions may attenuate TKV progression in ADPKD. While limited by sample size and intervention heterogeneity, this case series provides hypothesis-generating data to inform prospective controlled trials.

## Introduction

Autosomal dominant polycystic kidney disease (ADPKD) is the most common inherited kidney disorder and a leading genetic cause of end-stage renal disease. The disease is characterized by progressive cyst formation and enlargement, resulting in increasing total kidney volume (TKV), decline in renal function, and substantial morbidity. Mutations in *PKD1*, particularly truncating variants, are associated with more severe disease and earlier progression compared with non-truncating mutations (1).

Total kidney volume, especially when indexed to height and interpreted within the Mayo imaging classification framework, is a validated biomarker of disease progression risk in ADPKD. In natural history cohorts, TKV typically increases steadily over time, and spontaneous downward shifts in TKV are not a known feature of the disease (1). As a result, interventions capable of modifying volumetric trajectory remain of considerable clinical interest.

Emerging evidence suggests that cystic epithelial cells exhibit altered metabolic programming, including increased glycolytic flux and dysregulated energy-sensing pathways (2). Preclinical studies across multiple rodent and feline PKD models have demonstrated that ketogenic dietary interventions, fasting, and administration of *β*-hydroxybutyrate (BHB) are disease-modifying, reduce cyst burden, and slow or partially reverse disease progression (3–6). These findings suggest that metabolic reprogramming may represent a therapeutic target in ADPKD.

Early clinical data are beginning to translate these findings into human populations (7). Short-term pilot studies of ketogenic dietary interventions in ADPKD have demonstrated feasibility and induction of sustained ketosis, increased renal function and reduced kidney volume (8, 9). In addition to dietary approaches, a ketone- and citrate-based medical food combining exogenous *β*-hydroxybutyrate with alkalinizing citrate (KetoCitra^®^) has been developed to support metabolic interventions in kidney disease. Real-world outcomes from a structured ketogenic nutrition program including the use of KetoCitra^®^ in ADPKD have recently been reported, indicating favorable metabolic changes and stabilization or improvement of clinical parameters, including kidney function, body weight, pain, and medication use, in participating individuals (10–12). Interim results from an ongoing controlled clinical study evaluating ketone- and citrate-based supplementation in combination with nutritional changes have also suggested short-term reductions in kidney volume, although full peer-reviewed data are pending (13). Finally, patient communities worldwide have reported numerous real-world experiences suggesting potential benefits of metabolic approaches.

Despite these advances, longitudinal imaging trajectories in individuals implementing metabolic strategies remain limited in the peer-reviewed literature, and such approaches are not yet widely incorporated into routine clinical management. Systematically documented case-level imaging data may therefore help bridge this gap by providing insight into volumetric trajectory changes while informing the design of prospective controlled trials.

Here, we present a retrospective case series of four individuals with genetically confirmed truncating *PKD1* variants who implemented metabolic interventions of varying intensity, including carbohydrate restriction, intermittent fasting, and KetoCitra^®^ supplementation, for much longer periods of time than in currently available controlled studies. Serial imaging studies were re-analyzed to assess changes in TKV, liver volume, and Mayo imaging classification over time. Across cases, downward shifts in Mayo imaging trajectory were observed during intervention periods, accompanied by stabilization or reduction in TKV and preserved kidney function. These observations provide hypothesis-generating data to inform the design of prospective controlled studies evaluating metabolic approaches in ADPKD.

## Results

This retrospective case series included four individuals characterized by relatively long-term and continuous use of KetoCitra^®^ and availability of detailed clinical and imaging data. All four had genetically confirmed truncating *PKD1* variants (three male, one female; ages 33–71). The duration of metabolic intervention ranged from 6 months to approximately 4 years. Imaging studies obtained as part of routine clinical care were re-analyzed to determine TKV, liver volume, and Mayo imaging classification. Longitudinal kidney function was assessed using laboratory-reported estimated glomerular filtration rate (eGFR). Individual clinical courses are described below.

### Case 1

**Figure 1:**
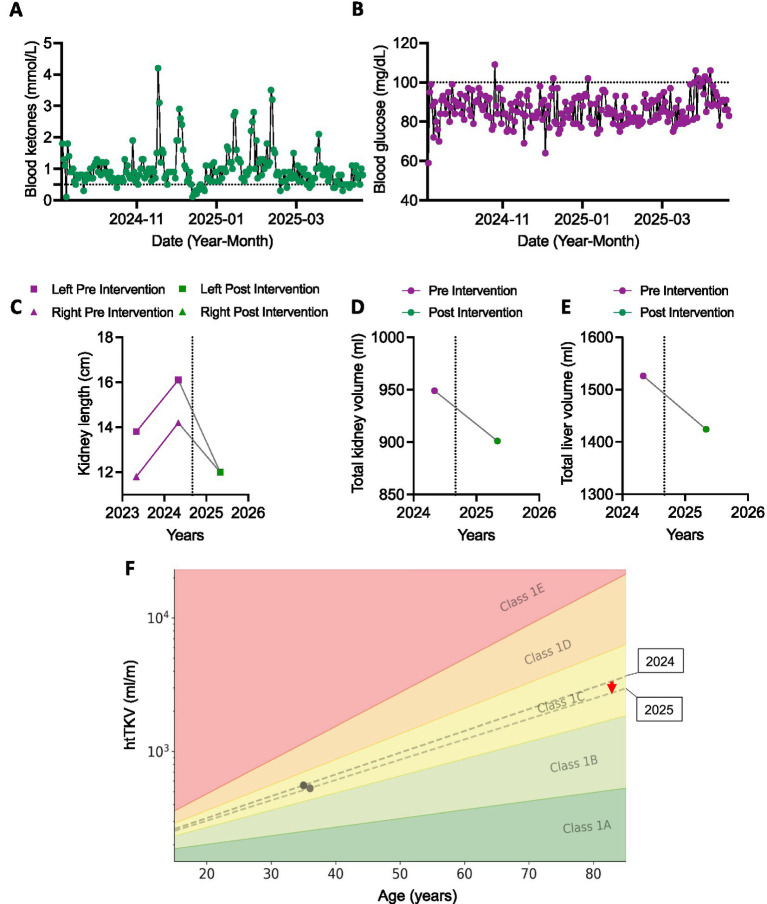
Metabolic and imaging data for Case 1. **(A)** Blood β-hydroxybutyrate measurements during the intervention period. Values above the dashed horizontal line (>0.5 mmol/L) are considered consistent with nutritional ketosis. **(B)** Values below the dashed horizontal line (<100 mg/dL) are consistent with normoglycemia during the intervention period. **(C)** Renal ultrasound images showing kidney length measurements before and after the intervention period (2023–2025). MRI-based volumetric analysis using TRACEORG showing **(D)** left and right kidney volumes, total kidney volume and **(E)** total liver volume at pre-intervention (April 2024) and post-intervention (April 2025) timepoints. **(F)** Mayo imaging classification trajectory over time. The dotted black lines in C-E indicate the start of intervention in September of 2024.

Case 1 is a 36-year-old male with a genetically confirmed truncating *PKD1* variant (c.6994_7000del; p. Ala2332Trpfs*7) and a diagnosis of ADPKD and polycystic liver disease. At baseline, Mayo imaging classification was 1C. The intervention period spanned 9 months (September 2024–May 2025). The participant implemented a sustained ketogenic dietary pattern with nutritional ketosis [blood β-hydroxybutyrate (BHB) > 0.5 mmol/L; blood glucose <100 mg/dL] and intermittent fasting, in conjunction with daily exogenous BHB and citrate (KetoCitra^®^) supplementation (Figures 1A,B and Supplementary Figure 1A).

Renal ultrasound suggested kidney enlargement prior to intervention (2023–2024), followed by a reduction in kidney length after the intervention period (Figure 1C). MRI-based volumetric analysis using 3D Slicer (Supplementary Figure 1B) and TRACEORG (Figure 1D) demonstrated a reduction in total kidney volume from 949 to 901 mL over the 9-month interval. Liver volume demonstrated a similar downward trend (Figure 1E). Mayo classification remained within Class 1C, with a downward shift within the classification range (Figure 1F). eGFR remained within the normal range throughout the intervention period and lipid parameters were consistent with a lean-mass hyper-responder phenotype (14, 15), and coronary artery calcium scoring was 0 (data not shown). Body weight remained largely stable, with only a 1.2 lb. decrease during the intervention period (145.0–143.8 lb.).

Overall, this case demonstrates reduction in total kidney volume with preserved kidney function during the intervention period, with imaging findings trending toward the lower-risk boundary within Mayo Class 1C.

### Case 2

Case 2 is a 40-year-old male with a genetically confirmed truncating *PKD1* variant (c.5014_5015del; p. Arg1672Glyfs*98) and a diagnosis of ADPKD without polycystic liver disease. Imaging and laboratory data were available from 2021 through 2025.

**Figure 2:**
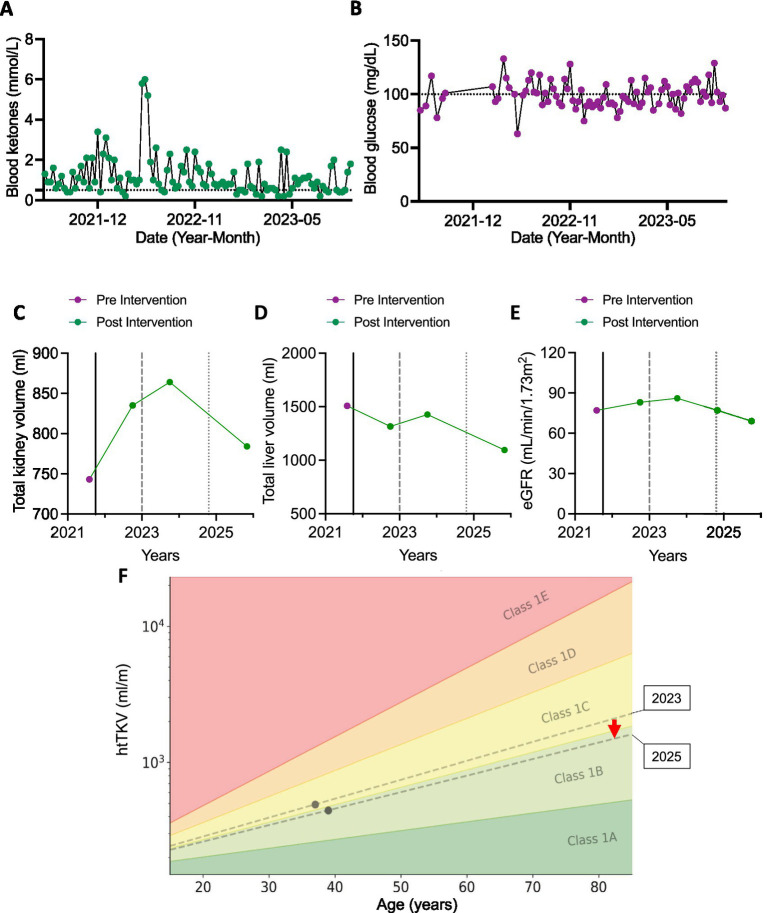
Imaging and clinical data for Case 2. **(A)** Blood β-hydroxybutyrate measurements during the intervention period. Values above the dashed horizontal line (>0.5 mmol/L) are considered consistent with nutritional ketosis. **(B)** Values below the dashed horizontal line (<100 mg/dL) are consistent with normoglycemia during the intervention period. **(C)** Total kidney volume measured by MRI-based volumetric analysis using TRACEORG across available imaging timepoints (2021–2025). **(D)** Total liver volume measured using TRACEORG across the same timepoints. **(E)** Estimated glomerular filtration rate (eGFR) values reported during routine clinical follow-up between 2021 and 2025. **(F)** Mayo imaging classification trajectory over time based on height-adjusted total kidney volume and age. The solid vertical lines in C-E indicate the initiation of ketogenic metabolic nutrition, the dashed vertical lines indicate the addition daily KetoCitra^®^ intake, and the dotted vertical lines indicates the addition of fasting.

The participant adopted the intervention in stages, beginning with a sustained ketogenic dietary pattern with nutritional ketosis [blood β-hydroxybutyrate (BHB) > 0.5 mmol/L; blood glucose <100 mg/dL] in late 2021 (Figures 2A,B), followed by daily KetoCitra^®^ use in early 2023 and fasting in late 2024. Between 2021 and 2023, MRI-based volumetric analysis using TRACEORG demonstrated progressive enlargement of total kidney volume (Figure 2C). Subsequent imaging in 2025 demonstrated a reduction in total kidney volume relative to peak 2023 measurements (864–784 mL) (Figure 2C).

Liver volume demonstrated variability across timepoints, with a lower value observed in 2025 compared with prior measurements (Figure 2D). eGFR ranged between 69 and 86 mL/min/1.73 m^2^ between 2021 and 2025 without sustained progressive decline (Figure 2E). Mayo imaging classification shifted from Class 1C at baseline toward Class 1B between 2023 and 2025 (Figure 2F). The participant reported an initial weight loss of >10 lb. (179–168 lb.) shortly after starting nutritional ketosis in 2021, followed by weight regain and stabilization around 170 lb. for the remainder of the intervention period (Supplementary Figure 2A). The patient attributed this to learning how to implement the approach appropriately and sustainably.

Overall, this case demonstrates reduction in total kidney volume with preserved kidney function during the observation period, accompanied by a shift toward a lower Mayo imaging classification.

### Case 3

Case 3 is a 33-year-old female with a genetically confirmed synonymous (splice-altering) *PKD1* variant (c.12048C > T; *p*.=) and a diagnosis of ADPKD and polycystic liver disease. At baseline, Mayo imaging classification was 1B. Serial imaging and laboratory data were available from 2022 through 2025.

Following diagnosis in 2019, the participant intermittently experimented with carbohydrate restriction and inconsistent use of KetoCitra^®^ (after its availability in 2022) between 2019 and 2024. The participant reported a prior history of binge eating disorder, which influenced dietary consistency between 2019 and 2024. Consistent KetoCitra^®^ use and strict carbohydrate restriction (<25 g total carbohydrates per day) was implemented during the 6 months preceding the 2025 MRI (November 2024 to May 2025). The participant reported regularly entering ketosis during the intervention period, although ketone measurement data were not available.

**Figure 3:**
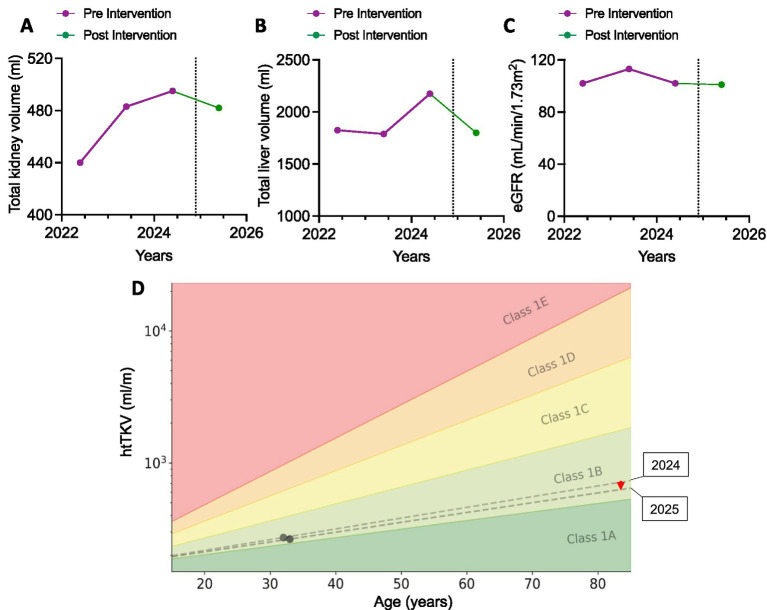
Imaging and clinical data for Case 3. **(A)** Total kidney volume measured by MRI-based volumetric analysis using TRACEORG across available imaging timepoints (2022–2025). **(B)** Total liver volume measured using TRACEORG across available imaging timepoints. **(C)** Estimated glomerular filtration rate (eGFR) values reported during routine clinical follow-up between 2022 and 2025. **(D)** Mayo imaging classification trajectory over time based on height-adjusted total kidney volume and age. The dotted black lines in A–C indicate the start of intervention in November of 2025.

Between 2022 and 2024, MRI-based volumetric analysis demonstrated progressive enlargement of total kidney volume from 440 mL in 2022 to 495 mL in 2024 (Figure 3A). Imaging in 2025 demonstrated relative stabilization with a slight decrease to 482 mL (Figure 3A). Liver volume demonstrated variability across timepoints, with a decrease from 2,176 mL in 2024 to 1801 mL in 2025 (Figure 3B). eGFR remained within the normal range (>90 mL/min/1.73 m^2^) across all available timepoints (Figure 3C). Mayo classification remained within Class 1B, with a flatter trajectory relative to age in 2025 (Figure 3D). The participant reported minor weight gain during the study intervention (165–172 lb.).

Overall, this case demonstrates stabilization of total kidney volume following a six-month period of consistent carbohydrate restriction and regular use of KetoCitra^®^, with preserved kidney function and imaging findings remaining within Mayo Class 1B.

### Case 4

Case 4 is a 71-year-old male with a genetically confirmed truncating *PKD1* variant (c.12031C > T; p. Gln4011*) and a diagnosis of ADPKD and polycystic liver disease. Baseline imaging from 2017 demonstrated a total kidney volume of 2,095 mL and an eGFR of 55.3 mL/min/1.73 m^2^.

**Figure 4:**
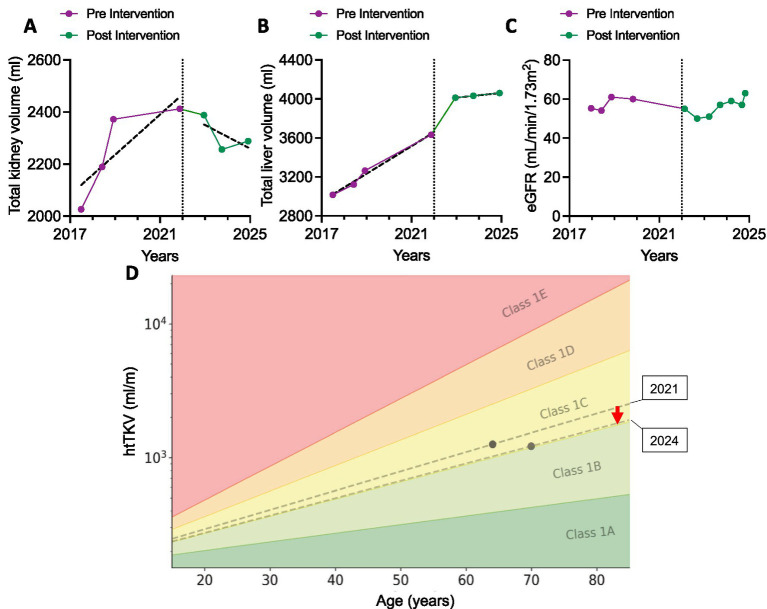
Imaging and clinical data for Case 4. **(A)** Total kidney volume measured by MRI-based volumetric analysis using TRACEORG across available imaging timepoints (2017–2024). **(B)** Total liver volume measured using TRACEORG across available imaging timepoints. **(C)** Estimated glomerular filtration rate (eGFR) values reported during routine clinical follow-up between 2017 and 2024. **(D)** Mayo imaging classification trajectory over time based on height-adjusted total kidney volume and age. The dotted black lines in A-C indicate the start of intervention in January 2022. Dashed black lines indicate linear regression.

Between 2017 and 2021, MRI-based volumetric analysis demonstrated progressive enlargement of total kidney and liver volumes (Figures 4A,B), with peak kidney volume reaching 2,412 mL in 2021 (estimated slope: +77.24 mL/year). Beginning in late 2021, the participant adopted consistent 16:8 time-restricted feeding and initiated daily KetoCitra^®^ use in early 2022 which continued during the depicted intervention period (3 years). Subsequent imaging between 2022 and 2024 demonstrated a reduction in total kidney volume to 2,288 mL (estimated slope: −47.20 mL/year) (Figure 4A).

Liver volume demonstrated progressive enlargement between 2017 and 2022 (estimated slope: +141.24 mL/year), followed by a reduced rate of increase between 2022 and 2024 (estimated slope: +24.45 mL/year) (Figure 4B). Estimated glomerular filtration rate (eGFR) was 55.3 mL/min/1.73 m^2^ in 2017 and measured 63 mL/min/1.73 m^2^ in 2024 without sustained decline during the observation period (Figure 4C). Mayo imaging classification shifted from mid-Class 1C toward the 1B/1C boundary during the intervention interval (Figure 4D). The participant reported a gradual decrease in body weight from 190 lb. in 2021 to 176 lb. in 2025 during the intervention period (Supplementary Figure 2B).

Overall, this case demonstrates reduction in total kidney volume with preserved kidney function during the observation period, accompanied by a shift toward a lower Mayo imaging risk category.

#### Dietary composition and ketosis

Dietary composition data were collected retrospectively through follow-up questionnaires and available dietary tracking records (Supplementary Table 2). Detailed tracking data was available for Cases 1–3. Participants generally reported carbohydrate restriction targeting <25–50 g/day with high-fat ketogenic dietary patterns. Reported macronutrient distributions, when available, were consistent with nutritional ketosis and ranged approximately from 5 to 6% carbohydrates, 17–20% protein, and 75–77% fat of total kilocalories. Protein intake varied across individuals and ranged from approximately 0.6–2.0 g/kg/day where estimations were available. Because dietary intake was self-reported and not prospectively standardized, these estimates should be interpreted cautiously. The reported dietary compositions were generally consistent with nutritional ketosis. Continuous blood ketone measurements were available for Cases 1 and 2 and supported sustained ketosis during the intervention period. Case 3 also reported regular blood ketone measurements consistent with ketosis (data not available), whereas Case 4 reported no detectable ketones on routine urine testing but did not measure blood ketone levels.

#### Reported outcomes, tolerability, and safety

Patient-reported outcomes and tolerability data were collected retrospectively using follow-up questionnaires (Supplementary Table 3). Overall, participants reported the interventions to be feasible and generally well tolerated. Two participants described significant improvement in quality of life, including improvements in mental clarity, work productivity, sleep, food cravings, and perceived physical well-being, while the remaining participants reported either mild improvement or no major change in quality of life. Several participants specifically described the interventions as sustainable long term. No serious adverse events were reported in the available clinical records. Reported adverse effects were generally mild and variable across individuals and included transient “keto flu”-like symptoms, gastrointestinal symptoms associated with KetoCitra^®^ intake, occasional muscle cramps, and a single reported episode of hypoglycemia symptoms associated with alcohol intake and hot tub exposure. No participant reported discontinuing the intervention because of adverse effects. Available laboratory data did not demonstrate consistent evidence of worsening kidney function, electrolyte disturbances, metabolic acidosis, or liver dysfunction during the intervention periods. Lipid responses varied across individuals, including increases in LDL cholesterol in some cases, whereas other participants demonstrated stable or favorable lipid profiles. Formal safety assessment was not performed in this retrospective analysis.

## Discussion

In this retrospective case series, four individuals with genetically confirmed truncating *PKD1* variants implemented metabolic interventions of varying intensity, including carbohydrate restriction, intermittent fasting, and KetoCitra^®^ supplementation. Across cases, TKV was reduced relative to prior growth trends, and kidney function remained preserved over the observation intervals. One participant transitioned from Mayo Class 1C to 1B, while the remaining three demonstrated downward shifts in trajectory within their existing Mayo classifications. Because TKV typically shows progressive upward trajectories in natural history cohorts, the decrease of TKV in all four subjects is notable. In all cases the intervention period followed a phase of documented volumetric growth, allowing comparison of trajectories before and after implementation of metabolic strategies. Notably, one participant (Case 2) adopted the intervention in stages and specifically attributed the later incorporation of intermittent fasting to the observed improvements in kidney volume, highlighting the possibility that fasting may represent an important contributing component of metabolic therapy in some individuals.

The observations reported here are broadly consistent with a growing body of literature suggesting that metabolic interventions may influence cystic disease biology in ADPKD (2). Preclinical studies across multiple rodent and feline PKD models have demonstrated that ketogenic dietary interventions, fasting, and administration of β-hydroxybutyrate reduce cyst burden and slow disease progression (3, 5, 6, 16). Early human studies have demonstrated feasibility of ketogenic metabolic therapy and induction of sustained ketosis in individuals with ADPKD, with preliminary signals suggesting potential effects on kidney volume and metabolic parameters (8, 9). In addition, real-world outcomes from structured ketogenic nutrition programs have recently been reported, and interim results from an ongoing controlled clinical study evaluating KetoCitra^®^ supplementation in combination with nutritional changes have suggested short-term reductions in kidney volume (10–12). While these emerging data require confirmation, the present case series aligns with this developing evidence base.

Changes in liver volume were also observed across several cases. Interpretation of hepatic volumetric changes is complex. In individuals without diagnosed polycystic liver disease, reductions in measured liver volume may reflect changes in hepatic glycogen content associated with carbohydrate restriction and ketogenic metabolism, as glycogen depletion is known to reduce liver volume (17, 18). In individuals with polycystic liver disease, cyst burden may also contribute to volumetric changes. TRACEORG-based cyst segmentation suggested fewer hepatic cysts following intervention in some cases (data not shown), although this observation cannot be interpreted conclusively given the limitations of automated segmentation and the retrospective nature of the data. Consequently, the relative contribution of glycogen depletion versus potential changes in cyst burden remains uncertain.

Ketogenic diets have previously been associated with an elevated risk of kidney stones, particularly those of calcium oxalate and uric acid (19). This could be a concern since individuals with ADPKD already have an elevated kidney stone risk and since cell injury from renal tubular microcrystals has been shown to accelerate PKD progression in mechanistic, preclinical studies (4, 5). All four cases supplemented with KetoCitra^®^ which, besides supporting ketosis, is designed to lower the risk of forming kidney stones and renal tubular microcrystals, particularly calcium oxalate and uric acid crystals/stones. This is due to (1) KetoCitra’s citrate content to help normalize urinary citrate levels, (2) it is alkaline base content to help normalize urinary pH, (3) and its content of calcium and magnesium to decrease the absorption of oxalate from diet. None of the four cases experienced kidney stones during their intervention.

This study has several important limitations. It is retrospective, includes a small number of self-selected individuals, and lacks a control group. The metabolic interventions were heterogeneous and not standardized, and imaging studies were obtained as part of routine clinical care rather than within a prospective protocol. Potential sources of bias include selection bias, regression to the mean, and variability in imaging acquisition and segmentation.

Changes in body weight may also represent an important potential confounding factor in the interpretation of volumetric outcomes. Obesity and visceral adiposity have previously been associated with accelerated ADPKD progression, and reductions in adiposity could plausibly influence cyst growth trajectories independently of ketosis itself. However, none of the participants in this series were obese at baseline, and overall weight changes were relatively modest. In addition, the relationship between weight change and TKV response was not consistent across cases. One participant maintained largely stable body weight despite TKV reduction, another demonstrated minor weight gain during the intervention period, and a third experienced early weight loss followed by prolonged weight stabilization prior to the later reduction in TKV. Only one participant demonstrated gradual long-term weight loss that temporally paralleled volumetric improvement. These observations suggest that weight loss alone is unlikely to fully explain the imaging findings, although its potential contribution cannot be excluded in this retrospective analysis.

Accordingly, these findings should be considered exploratory and hypothesis-generating. Nevertheless, the observation that all four individuals demonstrated stabilization or reduction in TKV during periods of metabolic intervention warrants further investigation, particularly given the typically progressive nature of ADPKD.

In summary, four individuals with truncating *PKD1* variants demonstrated downward shifts in Mayo imaging trajectory during periods of metabolic intervention, accompanied by stabilization or reduction in TKV and preserved kidney function. These observations are consistent with preclinical and emerging clinical evidence suggesting that metabolic approaches may be disease-modifying and influence structural disease progression in ADPKD and support further investigation in prospective controlled trials.

## Methods

### Study design and case identification

This retrospective descriptive case series includes four individuals with autosomal dominant polycystic kidney disease (ADPKD) who independently contacted the authors to share longitudinal clinical data following implementation of metabolic interventions. Cases were not consecutively enrolled and were not identified through systematic screening. Rather, individuals voluntarily provided de-identified medical records, laboratory data, and imaging studies after experiencing perceived changes in disease trajectory. All participants were additionally interviewed by the first author to collect detailed qualitative information regarding intervention adherence, patient-reported experiences, and potential adverse effects. None of the participants reported use of tolvaptan (Jynarque) before or during the intervention periods. Only Case 4 reported use of antihypertensive medication (perindopril, 4 mg/day) before and during the intervention period. All participants provided written informed consent for publication of de-identified clinical information.

### Metabolic interventions

Participants implemented individualized metabolic strategies aimed at promoting nutritional ketosis and/or ketone availability (see Supplementary Table 1 for details on duration, type of intervention, and patient characteristics). Approaches varied across cases and included combinations of ketogenic dietary patterns, intermittent fasting, and exogenous ketone supplementation. All four participants reported use of a ketone- and citrate-based medical food (KetoCitra^®^, Santa Barbara Nutrients, Santa Barbara, CA), which provides 5.3 g/d of β-hydroxybutyrate, 3.5 g/d of citrate, 600 mg/d potassium, 300 mg/d calcium, 250 mg/d magnesium and 51 mEq/d of total alkaline base to support nutritional ketosis, acid–base balance, to normalize urinary citrate, and to bind dietary oxalate. Besides supporting ketosis, KetoCitra^®^ is designed to lower the risk of forming kidney stones and renal tubular microcrystals, particularly calcium oxalate and uric acid. The degree of dietary carbohydrate restriction, fasting frequency, and adherence duration differed between participants. Interventions were self-directed and not standardized. The duration of intervention ranged from approximately 6 months to 3 years prior to the most recent available clinical evaluation. Additional retrospective follow-up questionnaires were administered to collect supplemental information regarding body weight, dietary composition, approximate macronutrient intake, patient-reported outcomes, tolerability, sustainability, and adverse effects associated with the interventions.

### Clinical and laboratory data

Longitudinal kidney function was assessed using creatinine-based eGFR values obtained from certified clinical laboratories as documented in medical records. eGFR values reflect calculations performed by the respective reporting institutions using standardized creatinine-based equations. Timepoints were irregular and correspond to routine clinical follow-up intervals.

### Imaging and volumetric analysis

MRI-based imaging studies were obtained as part of routine clinical care and may have been performed using varying acquisition parameters. Available abdominal imaging studies were re-analyzed in a blinded fashion by the authors using TRACEORG software (20). TRACEORG software was used for automated volumetric segmentation and Mayo classification assignment. Height-adjusted total kidney volume (htTKV) was calculated for each relevant timepoint. Mayo Clinic imaging classification was assigned based on htTKV and age using the automated classification output generated by TRACEORG.

3D Slicer software (3D Slicer)^1^ was used by the authors to verify total kidney volume (TKV) through semi-automated volumetric segmentation (21), with the Segmentation Wizard extension (author: Andrew Beers, Athinoula A. Martinos Center for Biomedical Imaging at Massachusetts General Hospital; available at: https://www.slicer.org/wiki/Documentation/Nightly/Extensions/SegmentationWizard, accessed 29 September 2025) (21). Using the Segment Editor module, renal boundaries were manually delineated across sequential slices with the paint tool to capture the full extent of kidney tissue, including cystic structures. Segmentation between annotated slices was interpolated using the “Fill between slices” function to generate a continuous kidney volume, followed by initialization to produce a three-dimensional reconstruction, which was visually inspected to confirm anatomical accuracy. Volumetric measurements were then obtained using the Segment Statistics module. The procedure was performed for each kidney separately, and volumes were summed to determine TKV at each imaging timepoint.

### Outcomes and statistics

Primary outcomes included longitudinal changes in eGFR and htTKV. Secondary outcomes included total liver volume, and Mayo imaging classification. Given the descriptive nature of this case series and small sample size, most analyses were limited to individual longitudinal trends without inferential statistical testing. Where sufficient longitudinal data points were available, linear regression analysis was performed to assess changes in total kidney volume (TKV) over time. Regression lines were fitted to individual patient data using time (in years) as the independent variable. In cases with adequate data coverage before and after intervention, analyses were performed separately for pre- and post-intervention intervals to estimate changes in trajectory. All analyses were conducted using GraphPad Prism (GraphPad Software, San Diego, CA, United States).

## Data Availability

The raw data supporting the conclusions of this article will be made available by the authors, without undue reservation.

## Footnotes

1
http://www.slicer.org

## Supplementary material

Supplementary Figure 1 Case 1 3D Fasting intervals and 3D Image Slicer Analysis. **(A)** Intermittent fasting pattern during the intervention period. **(B)** Longitudinal kidney volume measurements derived from MRI-based segmentation using 3D Slicer. Left and right kidney volumes are shown separately (left panel), along with total kidney volume (TKV) (right panel). Purple symbols represent pre-intervention measurements and green symbols represent post intervention measurements. The dotted vertical line indicates the initiation of the metabolic intervention.

Supplementary Figure 2 Case 2 and Case 4 body weight measurements. **(A)** Case 2 body weight measurements in pounds before and during the intervention period. For Case 2, the solid vertical line indicates initiation of ketogenic metabolic nutrition, the dashed vertical line indicates the addition daily KetoCitra^®^ intake, and the dotted vertical line indicates the addition of fasting. **(B)** Case 4 body weight measurements in pounds before and during the intervention period. For Case 4, the dotted vertical line indicates the initiation of the metabolic intervention (fasting and daily KetoCitra^®^ intake).
