# Supplementary material for: Halting cyst progression in ADPKD using long-term ketogenic metabolic therapy and supplementation with exogenous ketones and alkaline citrate—a case series
Source: Front Nutr. 2026 Jun 29;13:1843178. doi: 10.3389/fnut.2026.1843178 (PMC13357147; doi:10.3389/fnut.2026.1843178)
Supplement: Supplementary Figure 1 — Case 1 3D Fasting intervals and 3D Image Slicer Analysis. (A) Intermittent fasting pattern during the intervention period. (B) Longitudinal kidney volume measurements derived from MRI-based segmentation using 3D Slicer. Left and right kidney volumes are shown separately (left panel), along with total kidney volume (TKV) (right panel). Purple symbols represent pre-intervention measurements and green symbols represent post intervention measurements. The dotted vertical line indicates the initiation of the metabolic intervention. [file Image_1.pdf]

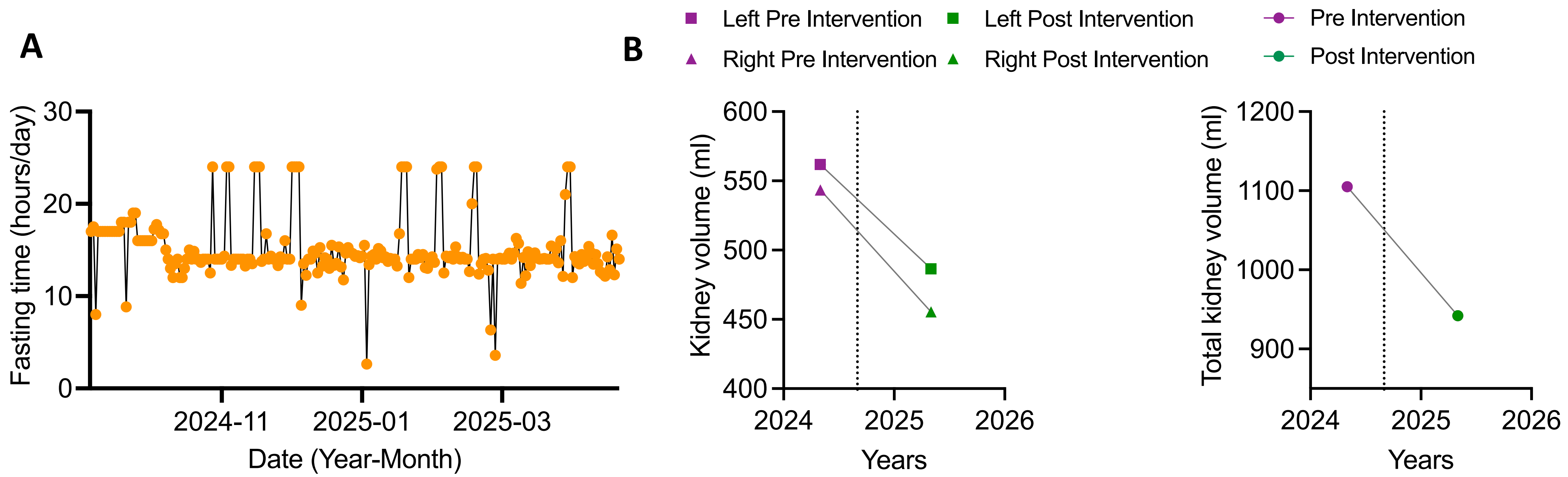

A
Fasting time (hours/day)
30
20
10
0
2024-11
2025-01
2025-03
Date (Year-Month)
B
Left Pre Intervention
Left Post Intervention
Right Pre Intervention
Right Post Intervention
Pre Intervention
Post Intervention
Kidney volume (ml)
600
550
500
450
400
2024
2025
2026
Years
Total kidney volume (ml)
1200
1100
1000
900
2024
2025
2026
Years
