# Supplementary figures and images for: Halting cyst progression in ADPKD using long-term ketogenic metabolic therapy and supplementation with exogenous ketones and alkaline citrate—a case series
Source: Front Nutr. 2026 Jun 29;13:1843178. doi: 10.3389/fnut.2026.1843178 (PMC13357147; doi:10.3389/fnut.2026.1843178)

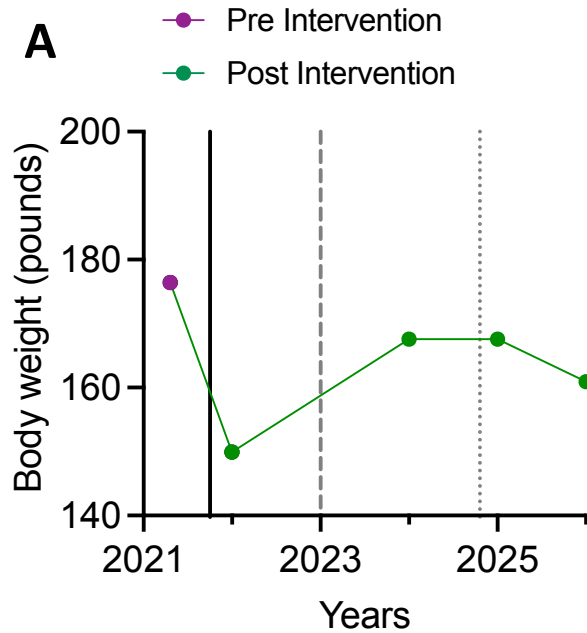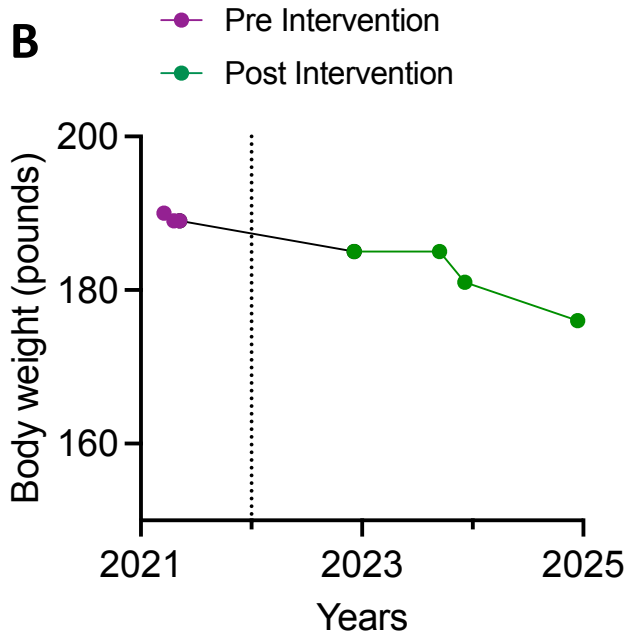

Supplement: Supplementary Figure 2 — Case 2 and Case 4 body weight measurements. (A) Case 2 body weight measurements in pounds before and during the intervention period. For Case 2, the solid vertical line indicates initiation of ketogenic metabolic nutrition, the dashed vertical line indicates the addition daily KetoCitra® intake, and the dotted vertical line indicates the addition of fasting. (B) Case 4 body weight measurements in pounds before and during the intervention period. For Case 4, the dotted vertical line indicates the initiation of the metabolic intervention (fasting and daily KetoCitra® intake). [file Image_2.pdf]
