# Supplementary material for: Halting cyst progression in ADPKD using long-term ketogenic metabolic therapy and supplementation with exogenous ketones and alkaline citrate—a case series
Source: Front Nutr. 2026 Jun 29;13:1843178. doi: 10.3389/fnut.2026.1843178 (PMC13357147; doi:10.3389/fnut.2026.1843178)
Supplement: Supplementary file 3 [file Supplementary_file_1.docx]

**Supplemental Figure 1. Case 1 3D Fasting intervals and 3D Image Slicer Analysis.** (A) Intermittent fasting pattern during the intervention period. (B) Longitudinal kidney volume measurements derived from MRI-based segmentation using 3D Slicer. Left and right kidney volumes are shown separately (left panel), along with total kidney volume (TKV) (right panel). Purple symbols represent pre-intervention measurements and green symbols represent post-intervention measurements. The dotted vertical line indicates the initiation of the metabolic intervention.

**Supplemental Figure 2. Case 2 and Case 4 body weight measurements.** (A) Case 2 body weight measurements in pounds before and during the intervention period. For Case 2, the solid vertical line indicates initiation of ketogenic metabolic nutrition, the dashed vertical line indicates the addition daily KetoCitra^®^ intake, and the dotted vertical line indicates the addition of fasting. (B) Case 4 body weight measurements in pounds before and during the intervention period. For Case 4, the dotted vertical line indicates the initiation of the metabolic intervention (fasting and daily KetoCitra^®^ intake).

**Supplemental Table 1: Basic characteristics of case series participants.**

| Parameter | Case 1 | Case 2 | Case 3 | Case 4 |
| --- | --- | --- | --- | --- |
| Age (y) / Sex | 36 / Male | 40 / Male | 33 / Female | 71 / Male |
| Body mass index (BMI) during study period* | Normal range | Normal range | Normal range | Normal range |
| Genetic variant | c.6994_7000del; p.Ala2332Trpfs*7 | c.5014_5015del; p.Arg1672Glyfs*98 | c.12048C>T; p.= | c.12031C>T; p.Gln4011* |
| Blood pressure during study period** | Normal range | Normal range | Normal range | Slightly elevated |
| Baseline eGFR (mL/min/1.73m²) | 109 | 77 | 102 | 55 |
| Presence of PLD | Yes | No | Yes | Yes |
| Baseline Mayo Classification | 1C | 1C | 1B | 1C |
| Intervention start /duration | 2024 / 9 months | 2021 / 4 years | 2024 / 6 months | 2022 / 3 years |
| Intervention details | Ketogenic Diet, Intermittent Fasting, KetoCitra^®^ Supplementation | Ketogenic Diet, Intermittent Fasting, KetoCitra^®^ Supplementation | Ketogenic Diet, Intermittent Fasting, KetoCitra^®^ Supplementation | Intermittent Fasting, KetoCitra^®^ Supplementation |
| Tolvaptan (Jynarque) use during study period | No | No | No | No |

*PLD = Polycystic Liver Disease*

**Normal BMI defined as 18.5–24.9 kg/m²*

**** *Normal blood pressure is defined as systolic <120 mmHg and diastolic <80 mmHg and slightly elevated is defined as systolic >120 mmHg and diastolic <80 mmHg.*

**Supplemental Table 2: Dietary composition**

| Macronutrient Information | Case 1 | Case 2 | Case 3 | Case 4 |
| --- | --- | --- | --- | --- |
| Approximate carbohydrate target (g/day) | \| \| <50 \| \| --- \| \| \| --- \| --- \| | <50 | <30–50 | \|  \| \| --- \|  \| Carbohydrate restriction reported; exact intake unavailable \| \| --- \| |
| Approximate carbohydrate intake (%) | 5 | 5 | 5 | Not available |
| Approximate protein intake (%) | 20 | 20 | 17.1 | Not available |
| Approximate fat intake (%) | 75 | 75 | 77.1 | Not available |
| Approximate protein intake (g/kg/day) | 1.8–2.0 | ~0.6 | 1.0–2.6 | Not available |
| Notes | Detailed tracking with Cronometer | Detailed tracking with Cronometer | Detailed tracking with Cronometer | Detailed tracking unavailable |

**Supplemental Table 3: Reported outcomes, tolerability, and safety**

| Outcomes Questions | Case 1 | Case 2 | Case 3 | Case 4 |
| --- | --- | --- | --- | --- |
| Quality of life impact | \| Significant improvement \| \| --- \| | Significant improvement | Somewhat improved | \|  \| \| --- \|  \| No major change \| \| --- \| |
| Sustainability | Sustainable | Sustainable | Sustainable with flexibility | \|  \| \| --- \|  \| Very easy to maintain \| \| --- \| |
| Main perceived benefits | Lifestyle change, stopped alcohol | Mental clarity, productivity, sleep | Reduced cravings, kidney discomfort improved | Convenience during travel |
| Reported adverse effects | None reported | Early keto flu | Occasional nausea/headaches, one hypoglycemia episode | Mild GI symptoms |
